# Measurement of gene amplifications related to drug resistance in *Plasmodium falciparum* using droplet digital PCR

**DOI:** 10.1186/s12936-021-03659-5

**Published:** 2021-02-28

**Authors:** Suttipat Srisutham, Kanokon Suwannasin, Rungniran Sugaram, Arjen M. Dondorp, Mallika Imwong

**Affiliations:** 1 Department of Clinical Microscopy, Faculty of Allied Health Sciences, Chulalongkorn University, Bangkok, Thailand; 2 Mahidol-Oxford Tropical Medicine Research Unit, Faculty of Tropical Medicine, Mahidol University, Bangkok, Thailand; 3 Division of Vector Borne Diseases, Department of Disease Control, Ministry of Public Health, Nonthaburi, Thailand; 4 Centre for Tropical Medicine and Global Health, Nuffield Department of Medicine, University of Oxford, Oxford, UK; 5 Department of Molecular Tropical Medicine and Genetics, Faculty of Tropical Medicine, Mahidol University, Bangkok, Thailand

**Keywords:** ddPCR, *Plasmodium falciparum*, *pfmdr1*, *pfplasmepsin2*, *pfgch1*

## Abstract

**Background:**

Copy number variations (CNVs) of the *Plasmodium falciparum multidrug resistance 1* (*pfmdr1*), *P. falciparum plasmepsin2* (*pfplasmepsin2*) and *P. falciparum GTP cyclohydrolase 1* (*pfgch1*) genes are associated with anti-malarial drug resistance in *P. falciparum* malaria. Droplet digital PCR (ddPCR) assays have been developed for accurate assessment of CNVs in several human genes. The aim of the present study was to develop and validate ddPCR assays for detection of the CNVs of *P. falciparum* genes associated with resistance to anti-malarial drugs.

**Methods:**

A multiplex ddPCR assay was developed to detect the CNVs in the *pfmdr1* and *pfplasmepsin2* genes, while a duplex ddPCR assay was developed to detect CNV in the *pfgch1* gene. The gene copy number (GCN) quantification limit, as well as the accuracy and precision of the ddPCR assays were determined and compared to conventional quantitative PCR (qPCR). In order to reduce the cost of testing, a multiplex ddPCR assay of two target genes, *pfmdr1* and *pfplasmepsin2*, was validated. In addition, the CNVs of genes of field samples collected from Thailand from 2015 to 2019 (n = 84) were assessed by ddPCR and results were compared to qPCR as the reference assay.

**Results:**

There were no significant differences between the GCN results obtained from uniplex and multiplex ddPCR assays for detection of CNVs in the *pfmdr1* and *pfplasmepsin2* genes (*p* = 0.363 and 0.330, respectively). Based on the obtained gene copy number quantification limit, the accuracy and percent relative standard deviation (%RSD) value of the multiplex ddPCR assay were 95% and 5%, respectively, for detection of the CNV of the *pfmdr1* gene, and 91% and 5% for detection of the CNV of the *pfplasmepsin2* gene. There was no significant difference in gene copy numbers assessed by uniplex or duplex ddPCR assays regarding CNV in the *pfgch1* gene (*p* = 0.276). The accuracy and %RSD value of the duplex ddPCR assay were 95% and 4%, respectively, regarding *pfgch1* GCN. In the *P. falciparum* field samples, *pfmdr1* and *pfplasmepsin2* GCNs were amplified in 15% and 27% of samples from Ubon Ratchathani, Thailand, while *pfgch1* GCN was amplified in 50% of samples from Yala, Thailand. There was 100% agreement between the GCN results obtained from the ddPCR and qPCR assays (κ = 1.00). The results suggested that multiplex ddPCR assay is the optional assay for the accurate detection of gene copy number without requiring calibration standards, while the cost and required time are reduced. Based on the results of this study, criteria for GCN detection by ddPCR analysis were generated.

**Conclusions:**

The developed ddPCR assays are simple, accurate, precise and cost-effective tools for detection of the CNVs in the *pfmdr1*, *pfplasmepsin2* and *pfgch1* genes of *P. falciparum*. The ddPCR assay is a useful additional tool for the surveillance of anti-malarial drug resistance.

## Background

Artemisinin-based combination therapy (ACT) is recommended as front-line treatment for *Plasmodium falciparum* malaria, which remains an important infectious disease in tropical regions. However, the emergence and spread of resistance to artemisinin-based combinations and related drugs have resulted in poor curative rates, especially in Southeast Asia [1–5]. Molecular surveillance is needed not only for the detection of mutations to the *P. falciparum kelch* gene, which are associated with artemisinin resistance [3], but also molecular markers associated with the efficacy of other anti-malarial drugs. An increase in the *P. falciparum multidrug resistance 1* (*pfmdr1*) GCN is associated with mefloquine resistance [6], while an increase in the *P. falciparum plasmepsin2* (*pfplasmepsin2*) GCN is associated with piperaquine resistance [7, 8]. Moreover, amplification of the *P. falciparum GTP cyclohydrolase 1* (*pfgch1*) GCN is linked to upregulation of the *P. falciparum dihydrofolate reductase* (*pfdhfr*) and *P. falciparum dihydropteroate synthase* (*pfdhps*) genes, which are associated with sulfadoxine-pyrimethamine resistance in Southeast Asia [9, 10].

Quantitative PCR (qPCR) assays are conventionally used to assess the copy number variations (CNVs) of genes related to drug resistance in *P. falciparum* malaria (i.e. *pfmdr1* [6], *pfplasmepsin2* [7], and *pfgch1* [11]). Alternatively, droplet digital PCR (ddPCR) technology was developed to measure CNVs and to provide highly precise measurements of the concentrations of target and reference genes in DNA samples [12, 13], as well as tolerance to PCR inhibitors, such as heparin [14], and to generate calibration curves to determine the GCNs of target sequences [15, 16]. The ddPCR assay is based on water-oil emulsion droplet technology used for detection and quantification of target gene [17]. The ddPCR reaction contains the ddPCR reagent, DNA samples, primers and fluorescent probe. All components are divided into around 15,000–20,000 droplets using the droplet generator. Each droplet may contain one, more than one or no copies of the DNA target [12, 13, 18]. After 40 cycles of the standard PCR reaction, DNA targets in each droplet are amplified and then analysed by a droplet reader. The DNA target concentration is calculated from the number of positive and negative droplets using Poisson statistics [12]. The ddPCR assay has been developed for accurate detection of CNVs in human genes associated with various human genetic diseases [19–21]. In addition, a ddPCR assay was developed and validated for the detection and quantification of *Plasmodium* species based on the *18S rRNA* gene sequence [22, 23], but this method has not yet been validated for the detection of the CNVs of genes associated with resistance to anti-malarial drugs.

In the present study, ddPCR assays were developed and validated for quantification of the CNVs of the *pfmdr1*, *pfplasmepsin2* and *pfgch1* genes, as well as for the molecular surveillance of the efficacy of anti-malarial drugs in field isolates. The ddPCR assays were used to detect the CNVs of the *pfmdr1*, *pfplasmepsin2* and *pfgch1* genes in field samples and validated against the results obtained with qPCR assays. A flowchart was generated including criteria for the detection of GCN with the novel ddPCR assays. Costs and time required for the ddPCR assays were discussed.

## Methods

### DNA samples

Development and validation of the ddPCR assays used DNA extracted from *P. falciparum* laboratory strains obtained from the Malaria Research and Reference Reagent Resource Center (American Type Culture Collection, Manassas, VA, USA). Parasite DNA from *P. falciparum* strain 3D7 (MRA-102), which carries single copies of the *pfmdr1* and *pfplasmespin2* genes, was used to develop and validate the ddPCR assays for the detection of the CNVs of these two genes. DNA samples of *P. falciparum* strain D6 originating from Sierra Leone, West Africa, which carries a single copy of the *pfgch1* gene [10], were used as single copy controls for the development and validation of ddPCR assays to detect the CNVs of the *pfgch1* gene. Two-fold serial dilutions of *P. falciparum* strains 3D7 (approximate 40,000 *P. falciparum* genome copies/ul based on the absolute quantification of *pfβtubulin* gene) and D6 (approximate 50,000 *P. falciparum* genome copies/ul based on the absolute quantification of *pfβtubulin* gene) were prepared and used to quantify the GCN, as well as to assess the accuracy and precision of the ddPCR assays.

*Plasmodium falciparum* strains 3D7 (isolated in Amsterdam), 7G8 (isolated in Brazil), D6 (isolated in Sierra Leone), D10 (isolated in Papua New Guinea), Dd2 (derived from cultivation), HB3 (isolated in Honduras), and W2 (isolated in Lao People’s Democratic Republic) obtained from the Malaria Research and Reference Reagent Resource Center (n = 7) were used to compare the CNVs obtained by the ddPCR and qPCR assays. In addition, the CNVs of the *pfmdr1*, *pfplasmepsin2* and *pfgch1* genes of field samples (n = 84) collected from patients with confirmed *P*. *falciparum* infections between 2015 and 2019 in Ubon Ratchathani (n = 60) and Yala (n = 24), Thailand, were determined. DNA samples were extracted using the QIAamp DNA Mini Kit (Qiagen, Hilden, North Rhine-Westphalia, Germany). DNA concentrations were measured using a Nanodrop™ spectrophotometer (Thermo Scientific, Willington, DE, USA). The study protocol was approved by the Ethical Review Committee of the Faculty of Tropical Medicine, Mahidol University (Bangkok, Thailand) (approval no. MUTM 2012-045-05).

### Development of the ddPCR assays

**Fig. 1:**
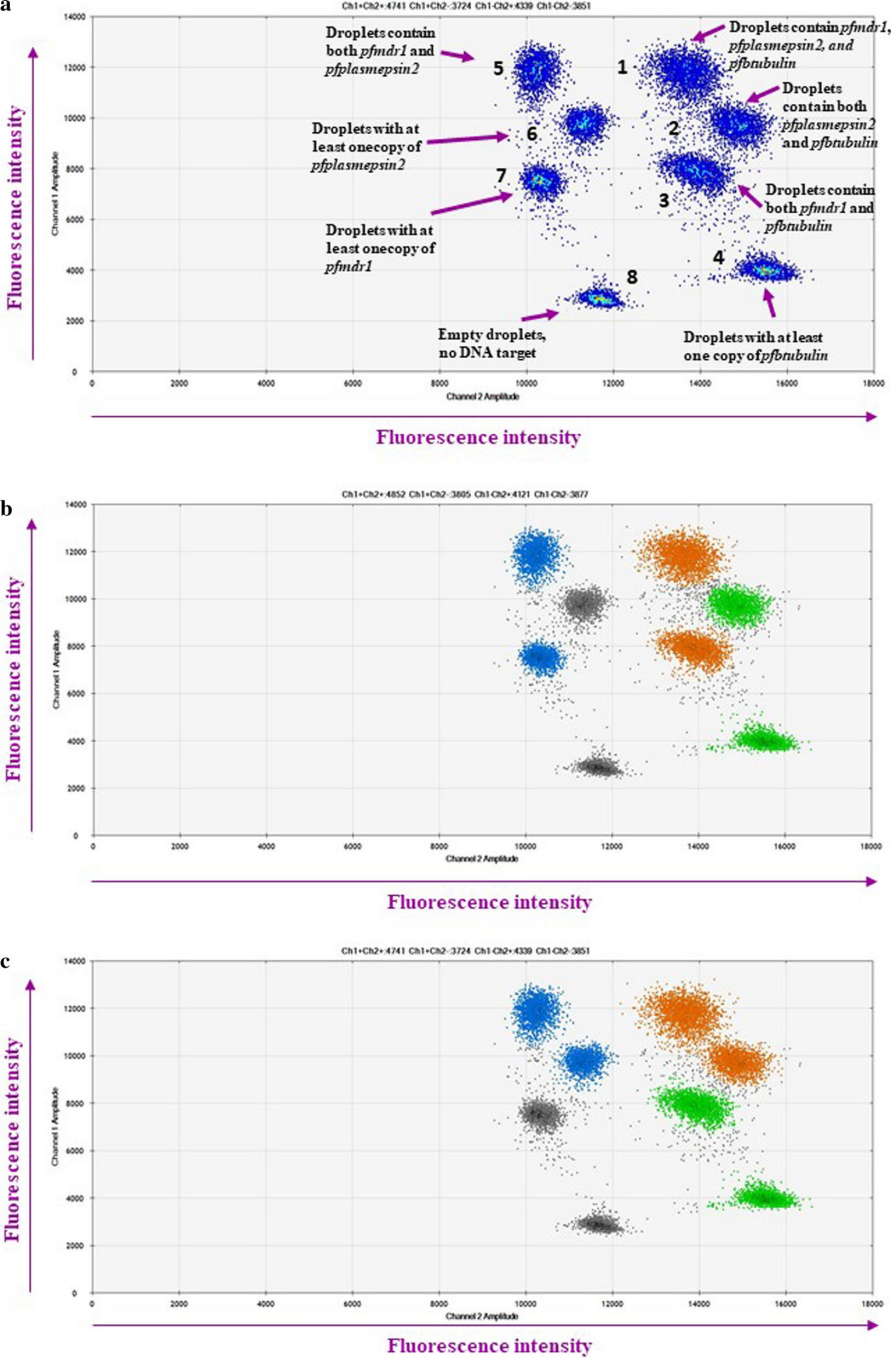
Two dimensional ddPCR amplitude plots of multiplex ddPCR assays. Heat map shows 8 clusters of droplets (**a**) including, droplets contain *pfmdr1*, *pfplasmepsin2*, and *pfbtubulin* (cluster 1), droplets contain both *pfplasmepsin2* and *pf-β-tubulin* (cluster 2), droplets contain both *pfmdr1* and *pf-β-tubulin* (cluster 3), droplets with at least one copy of *pf-β-tubulin* (cluster 4), droplets contain both *pfmdr1* and *pf-β-tubulin* (cluster 5), droplets with at least one copy of *pfplasmepsin2* (cluster 6), droplets with at least one copy of pfmdr1 (cluster 7), Empty droplets, no DNA target (cluster 8). Classification cluster of droplets for pfmdr1 copy number detection (**b**). Classification cluster of droplets for *pfplasmepsin2* copy number detection (**c**)

For the ddPCR assays, each 20 µL reaction contained 10 µL of ddPCR™ Supermix for Probes (Bio-Rad Laboratories, Hercules, CA, USA), 900 nM for each primer (1.8 µL of 10 µM of primer), 250 nM for each probe (0.5 µL of 10 µM probe) and 2 µL of DNA as a template. The primers and probes were previously designed for qPCR assays [6, 7, 11]. The *P. falciparum β tubulin* (*pfβtubulin)* gene was used as reference housekeeping gene. The ddPCR reaction was separated into 12,000–20,000 droplets using a QX200™ Droplet Generator (Bio-Rad Laboratories) and conducted using a T100™ Thermal Cycler (Bio-Rad Laboratories). During development of the ddPCR assays, a series of temperatures was tested to determine the optimal annealing temperature. Uniplex, duplex and multiplex ddPCR assays were developed to measure the CNVs of the *pfmdr1*, *pfplasmepsin2* and *pfgch1* genes. The optimal annealing temperature for the uniplex ddPCR assay of the *pfmdr1*, *pfplasmepsin2*, *pfgch1* and *pfβtubulin* genes was determined to be 56 °C (Additional file 1), while that for the duplex ddPCR assay for detection of *pfmdr1/pfβtubulin* and *pfplasmepsin2/pfβtubulin* genes was 58 °C and for the duplex ddPCR assay of the *pfgch1/pfβtubulin* genes, the optimal annealing temperature was 60 °C (Additional file 2). The optimal annealing temperature for the multiplex ddPCR assay of the *pfmdr1*/*pfplasmepsin2*/*pfβtubulin* genes was 60 °C (Fig. 1). For validation, the ddPCR assays were performed in triplicate. After amplification, the ddPCR data were read with the use of a QX200™ Droplet Reader (Bio-Rad Laboratories) and analysed using QuantaSoft™ Software version 1.7.4 (Bio-Rad Laboratories). At least 12,000 accepted droplets were analysed [24, 25]. Manual thresholds were applied to distinguish between positive and negative droplets. The fluorescence intensity thresholds were determined manually for each independent experiment using the using the clustering tool in the QuantaSoft™ Software. Positive and negative controls were included in each run. The GCN was calculated as the ratio of the concentration (copies/µL) of the target gene to that of the reference gene.

### Validation of ddPCR assays

Two-fold serial dilutions of *P*. *falciparum* strain 3D7 were prepared to quantify the CNVs of the *pfmdr1* and *pfplasmepsin2* genes, as well as validation of the accuracy and precision of the uniplex, duplex and multiplex ddPCR assays. Two-fold serial dilutions of *P. falciparum* strain D6 were prepared for validation of the uniplex and duplex ddPCR assays of the CNV of the *pfgch1* gene. The GCN was determined by three independent ddPCR runs. The accuracy of the ddPCR assays was calculated as % accuracy = 100 − %error and %error = the absolute difference between 1 and the GCN determined with the ddPCR assays. The precision of the ddPCR assays was calculated as the percent relative standard deviation (%RSD) = standard deviation/average × 100. Since the DNA samples might contain both *P*. *falciparum* and human DNA, the limit of the GCN, as determined with the ddPCR assays, was quantified based on the lambda (λ) value, which was calculated as λ = ln (number of negative droplets/numbers of accepted droplets). The limit of GCN quantification of the ddPCR assays is the range of the λ value providing a %RSD value of greater than 20% and %accuracy value of greater than 80% [25, 26]. In accordance with the guidelines of the Minimum Information for Publication of Quantitative Digital PCR Experiments [27], the GCN results of the *P. falciparum* reference strain were compared between the uniplex, duplex and multiplex ddPCR assays.

### Assessment of *pfmdr1*, *pfplasmespsin2* and *pfgch1* CNVs of *P. falciparum* reference strains and field isolates from Thailand

DNA samples from the *P. falciparum* reference strain (n = 7) and the *P. falciparum* isolates from Thailand (n = 84) were used to evaluate the ddPCR assays. The CNV results obtained by the ddPCR assays were compared with the results of the qPCR assays, as previously described [6, 7, 11].

### Statistical analysis

The GCN results of the *P. falciparum* reference strain, as determined with the uniplex, duplex and multiplex ddPCR assays, were compared using the independent samples median test with IBM SPSS Statistics for Windows, version 22.0 (IBM Corporation, Armonk, NY, USA). The kappa statistic was used to identify agreements between the GCN results obtained with the ddPCR assays and those obtained with the qPCR assays with the use of IBM SPSS Statistics for Windows, version 22.0.

## Results

### Development and validation of ddPCR assays for CNV measurements

#### Accuracy of the ddPCR assays

**Fig. 2:**
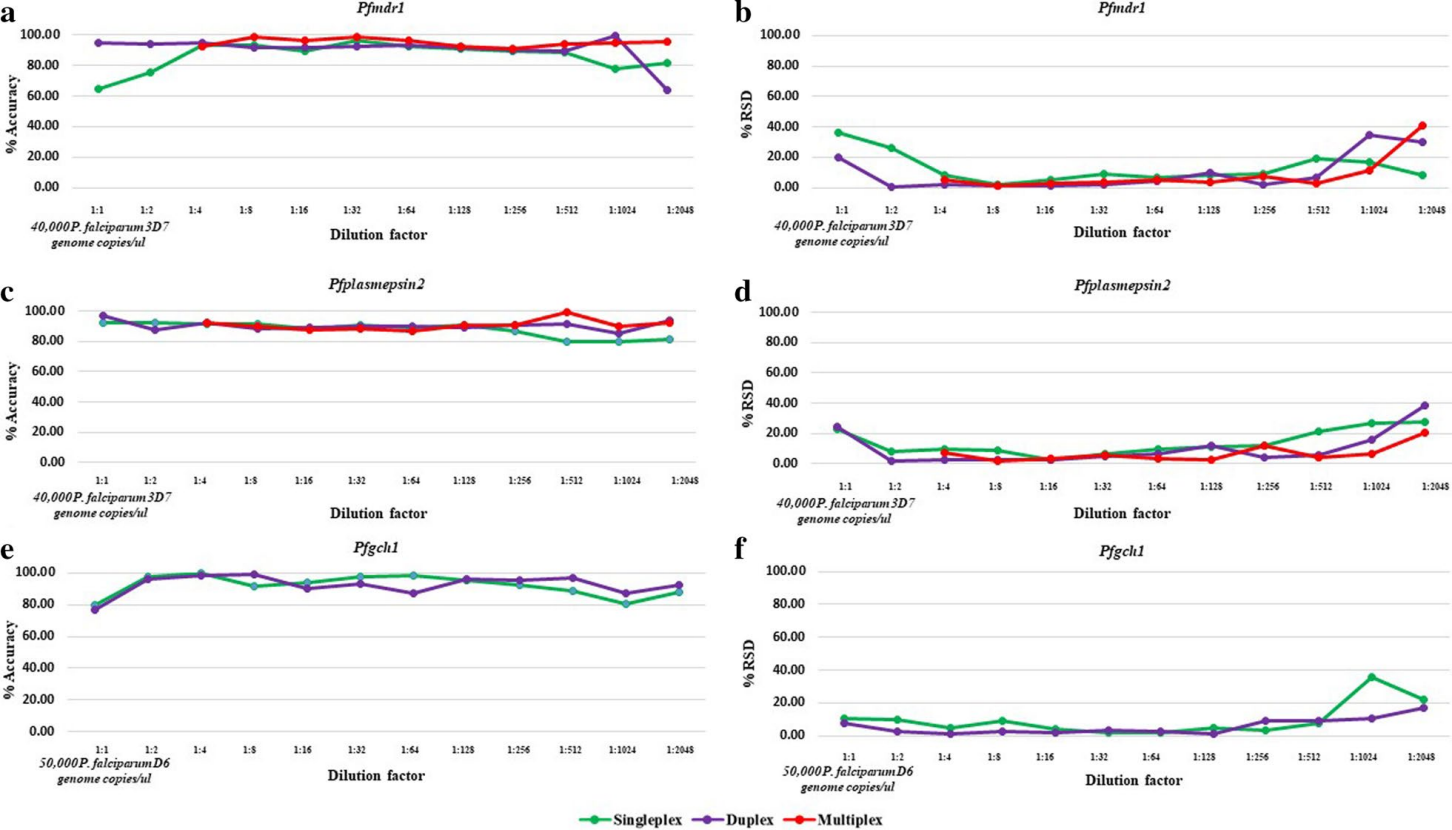
Limitation of gene quantification, accuracy and precision of ddPCR assays for *pfmdr1* copy number detection (**a**), *pfplasmepsin2* copy number detection (**b**), and *pfgch1* copy number detection (**c**)

As shown in Fig. 2, the accuracies of the uniplex, duplex and multiplex ddPCR assays were 65–96%, 64–99% and 91–99%, respectively, for measurement of the *pfmdr1* GCN, and 76–96%, 85–97% and 87–99%, respectively, for measurement of the *pfplasmepsin2* GCN. Meanwhile, the accuracies of the uniplex and duplex ddPCR assays for measurement of the *pfgch1* GCN were 80–100% and 77–99%, respectively.

#### Precision of ddPCR assays

As shown in Fig. 2, the %RSD values of the uniplex, duplex and multiplex ddPCR assays were 2–36%, 0–35% and 1–41 %, respectively, for detection of the *pfmdr1* GCN, and 3–28%, 2–39% and 2–21%, respectively, for detection of the *pfplasmepsin2* GCN. Meanwhile, the %RSD values of the uniplex and duplex ddPCR assays were 2–36% and 1–17% for detection of the *pfgch1* GCN.

### Limitation of GCN quantification

As shown in Table 1, the accepted range of λ values of the uniplex, duplex and multiplex ddPCR assays were 0.011–0.987, 0.005–2.178 and 0.003–0.842, respectively, for the *pfmdr1* GCN, and 0.010–1.870, 0.002–1.890 and 0.003–0.941, respectively, for the *pfplasmepsin2* GCN. The accepted range of λ values of the uniplex and duplex ddPCR assays were 0.006–1.915 and 0.003–1.877, respectively, for the *pfgch1* GCN. Based on the limitation of GCN quantification, the average accuracy and %RSD value of the multiplex ddPCR assay were 95% and 5%, respectively, for measurement of the *pfmdr1* GCN, and 91% and 5%, respectively, for measurement of the *pfplasmepsin2* GCN. The accuracy and %RSD value of the duplex ddPCR were 95% and 4%, respectively.

**Table 1:** Descriptive statistics of *P. falciparum mdr1, plasmepsin2*, and *gch1* CNVs based on the accepted lamda(λ) value of ddPCR assay of *pf-β-tubulin* gene

Statistic	*pfmdr1* CNVs			*pfplasmepsin2* CNVs			*pfgch1* CNVs	
	Singleplex	Duplex	Multiplex	Singleplex	Duplex	Multiplex	Singleplex	Duplex
Range	0.253	0.177	0.225	0.315	0.276	0.214	0.324	0.236
Minimum	0.812	0.817	0.861	0.814	0.738	0.813	0.868	0.926
Maximum	1.065	0.994	1.086	1.129	1.014	1.027	1.192	1.162
Mean	0.922	0.919	0.964	0.943	0.895	0.906	1.016	1.039
SD	0.061	0.040	0.061	0.085	0.057	0.057	0.078	0.065
Variance	0.004	0.002	0.004	0.007	0.003	0.003	0.006	0.004
%RSD	6.954	3.273	4.755	8.675	5.947	5.385	5.282	3.705
%Accuracy	92.240	92.135	95.047	93.304	89.533	90.638	95.118	94.876
Accepted lamda(λ) range	0.011–0.976	0.005–2.173	0.003–0.839	0.010–1.860	0.002–1.890	0.003–0.938	0.006–1.909	0.003–1.874

### Comparison between the uniplex, duplex and multiplex ddPCR assays

**Fig. 3:**
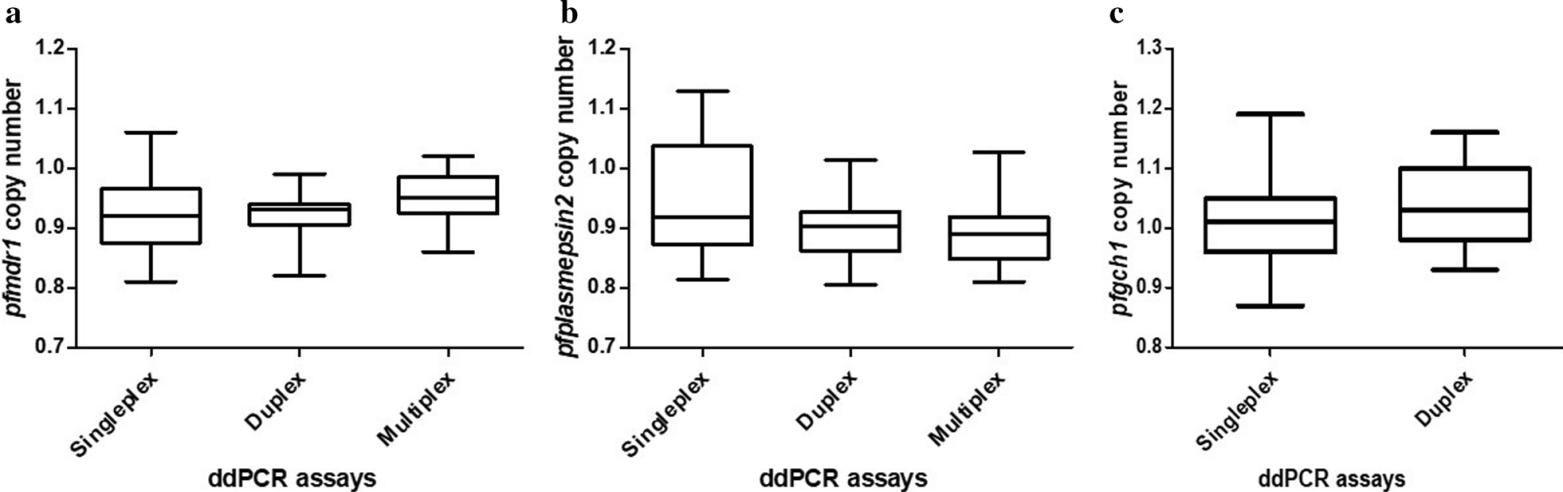
Whisker plots show median, maximum, and minimum of estimated *pfmdr1* (**a**), *pfplasmepsin2* (**b**), and *pfgch1* (**c**) copy number

Two-fold serial dilutions of DNA from *P*. *falciparum* strain 3D7 (4, 2, 1, 0.5, 0.25, 0.125, 0.0625, 0.03125, 0.015625, 0.0078125, 0.00390625 and 0.001953125 ng/µL) were used to compare the CNVs of the *pfmdr1* and *pfplasmepsin2* genes obtained from the uniplex, duplex and multiplex ddPCR assays. In addition, two-fold serial dilutions of DNA from *P. falciparum* strain D6 (8, 4, 2, 1, 0.5, 0.25, 0.125, 0.0625, 0.03125, 0.015625, 0.0078125 and 0.00390625 ng/µL) were used for comparison of the CNVs of the *pfgch1* gene obtained with the uniplex and duplex ddPCR assays. As shown in Fig. 3, there were no significant differences in the *pfmdr1* (*p* = 0.363) and *pfplasmepsin2* (*p* = 0.330) GCNs, as determined with the uniplex, duplex and multiplex ddPCR assays. In addition, there was no significant difference for detection of the *pfgch1* GCN between the uniplex and duplex ddPCR assays (*p* = 0.276).

### Standardized analytical workflow of ddPCR analysis for quantification of GCN

A flowchart, including validation criteria, for a standardized analytical workflow of ddPCR analysis was designed based on the observed limit of quantification of the optimal accuracy and precision. Control samples, as well as positive and negative controls, were included for each ddPCR assay. The accepted criteria used for the ddPCR assay are that the results of the negative control are negative, those of the positive single copy control are positive (ratio = 0.80–1.20) and those of the positive multiple copy control are positive (ratio > 1.20). Each ddPCR reaction contained at least 12,000 droplets. The GCN results were considered acceptable at a λ value of 0.003–0.800 for quantification of the *pfmdr1* and *pfplasmepsin2* genes with the multiplex ddPCR assay, and 0.003–1.900 for quantification of the *pfgch1* gene with the duplex ddPCR assay. GCNs were calculated as the ratio of the concentrations (copies/µL) of the target and references genes (single GCN ratio of 0.8–1.2 and multiple GCN ratio of > 1.20).

### Agreement of GCN results between the ddPCR and qPCR assays

**Fig. 4:**
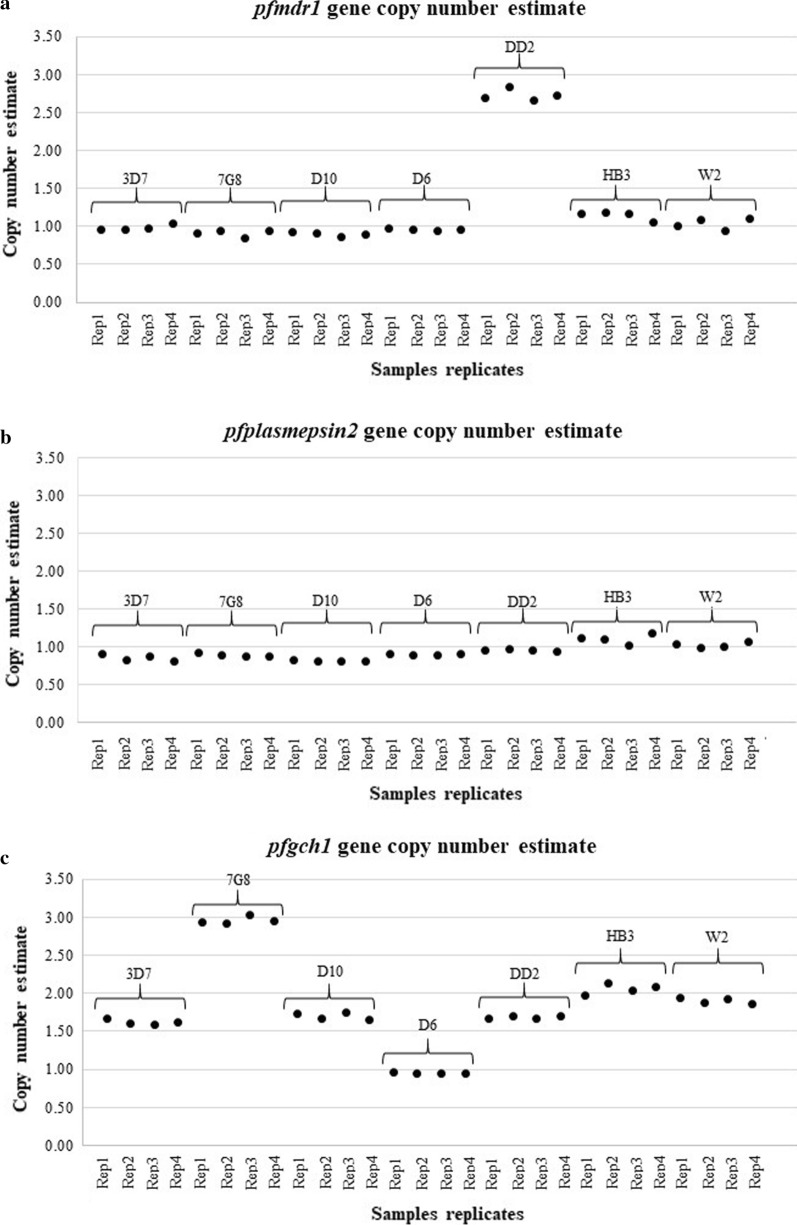
Genes copy number of *P. falciparum mdr1* (**a**), *plasmepsin2* (**b**), and *gch1* of reference strains estimated by ddPCR in replicates

DNA samples from the *P. falciparum* reference strain (n = 7) and *P. falciparum* strains 3D7, 7G8, D10, DD2, HB3, W2 and D6 were collected to compare the *pfmdr1*, *pfplasmepsin2* and *pfgch1* GCNs determined with the ddPCR and qPCR assays. The results showed 100% agreement between the ddPCR and qPCR assays (κ = 1) (Fig. 4, Additional file 3).

### CNVs of the *pfmdr1*, *pfplasmespsin2* and *pfgch1* genes of *P. falciparum* isolates from Thailand

**Fig. 5:**
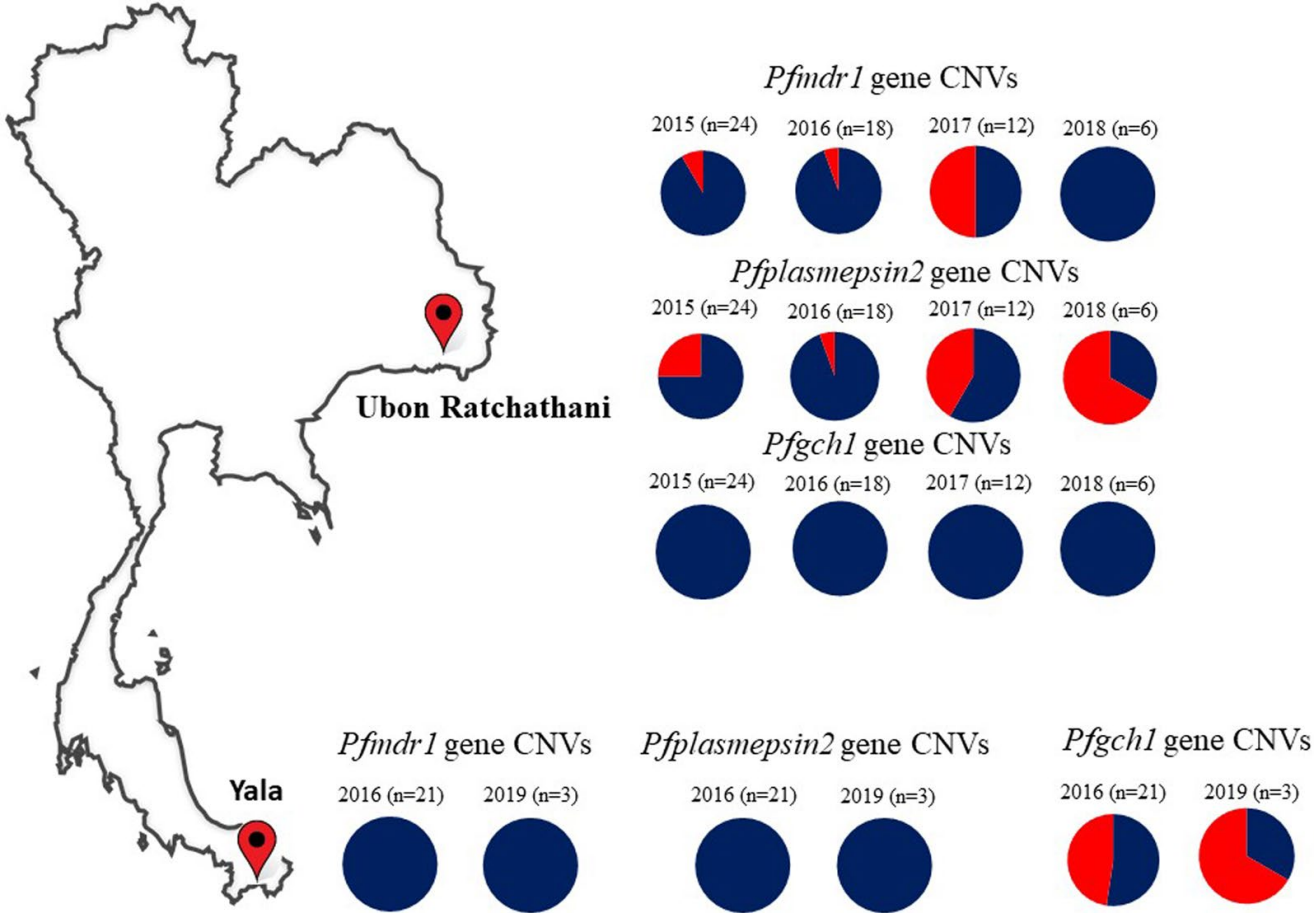
Prevalence of *pfmdr1*, *pfplasmepsin2*, and *pfgch1* gene amplification isolated from Ubon Ratchathani and Yala

The extracted DNA samples were determined the CNVs in the *pfmdr1*, *pfplasmespsin2* and *pfgch1* genes following the standardized analytical workflow obtained from this study. The results showed that the *pfmdr1* and *pfplasmepsin2* GCNs were amplified in 15% and 27% of samples from Ubon Ratchathani, Northeastern Thailand, suggested evidence of selection whereas no amplification in isolates from Yala, Southern Thailand. For the *pfgch1* GCN was amplified in 50% of samples from Yala, while no amplification in isolates from Ubon Ratchathani (Fig. 5, Additional file 4). Comparisons of the results of the ddPCR and qPCR assays were 100% in agreement for CNV assessments of the *pfmdr1*, *pfplasmepsin2* and *pfgch1* genes.

### Cost and turn‐around time of ddPCR assays

The costs of the uniplex, duplex and multiplex ddPCR assays to determine the CNVs of the *pfmdr1*, *pfplasmepsin2* and *pfgch1* genes were 10.40, 5.50 and 5.70 USD per sample, respectively. The turn-around times for the uniplex, duplex and multiplex ddPCR assays of 96 samples were12, 6 and 6 h, respectively.

## Discussion

The ddPCR has been suggested as the reliable alternative method for high-throughput GCNs quantification [12, 13]. Previously, ddPCR assay was developed for screening gene deletions and duplications in human genes such as Breast cancer type 1 susceptibility protein (BRCA1) which plays a significant role in carcinogenesis of breast and ovarian cancer [20], Leucine-rich repeats and immunoglobulin-like domains 1 (LRIG1) which may be determinants of breast cancer prognostic marker [28], and mitochondrial DNA (mtDNA) which varies during aging and disease progression [29]. In malaria, qPCR assay is the most commonly used assay for the identification of genes associated with anti-malarial drug resistance [6, 7, 11]. Here, ddPCR assays were developed and validated for accurate assessment of the CNVs of several *P. falciparum* resistance genes. GCNs estimated by the qPCR assay were measured based on exponential curves and calculated by the formula; (Ct of a target gene − Ct of a reference gene) of sample − (Ct of a target gene − Ct of a reference gene) of reference sample or = 2^−ΔΔCt^, while estimate with the ddPCR assays were measured based on the ratio of the absolute gene concentrations of the target and reference genes [13]. Therefore, GCNs of ddPCR were measured without requiring the reference sample.

**Fig. 6:**
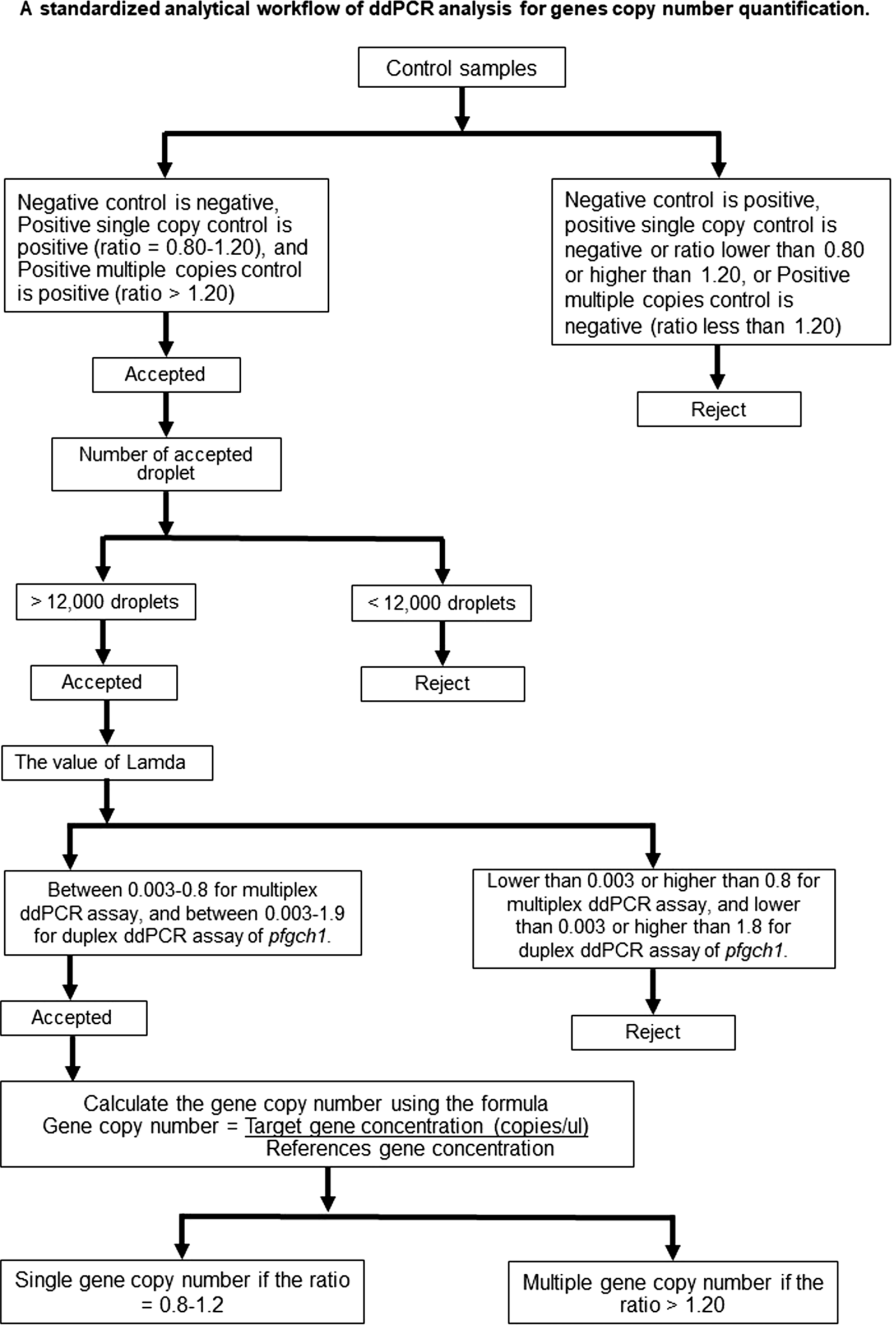
A standardized analytical workflow of ddPCR analysis used for genes copy number quantification

Although, the ddPCR assay was suggested for accurate GCNs quantification, previous study demonstrated that the accuracy and precision of GCNs quantification were reduced when using too high or too low concentration of the target genes [30]. Similarly, the results of the present study demonstrated that a higher or lower concentration of the target gene might affect the accuracy and precision of the ddPCR assays. As a consequence, optimal concentrations of the target genes are required for accurate detection of the GCNs. Here, an optimal DNA template was evaluated to accurately and precisely determine the GCN based on the λ value, which is estimated from the numbers of negative and accepted droplets generated by the ddPCR assays. So, the limit of quantification of the multiplex ddPCR assay of *pfmdr1* and *pfplasmepsin2* were based on the λ value which is between 0.003 and 0.8 and the limit of quantification of the duplex ddPCR assay is also based on the λ value which is between 0.003 and 1.9 for duplex ddPCR assay (Fig. 6).

To reduce the cost and turn-around time, multiplex ddPCR assays were developed for detection of both the *pfmdr1* and *pfplasmepsin2* genes in a single reaction. The results showed that there were no significant differences in the GCN assessments between the assays, favouring the multiplex, rather than the uniplex, ddPCR assay as the preferred method [27]. In, addition, a duplex ddPCR assay was also developed for the detection of the *pfgch1* GCN instead of the uniplex ddPCR assay. Although there was no significant difference between the GCNs results obtained from the singleplex and multiplex ddPCR assay, for the culture strain experiments, the *pfmdr1* and *pfplasmepsin2* copy number relative to *pfβtubulin* ratio is consistently below 1.0 with medians near 0.9 for all suggested that the results obtained from multiplex ddPCR would be further normalized with a number to provide more accurate value.

Compared to the uniplex ddPCR assay, use of the duplex ddPCR assay can reduce costs by 47% from 10.40 to 5.47 USD, while the estimated cost of qPCR is around 6.7 USD per sample. Duplex ddPCR assay can reduce the required assay time by 50% from 12 to 6 h. Moreover, the use of the multiplex ddPCR PCR assay to detect the *pfmdr1* and *pfplasmepsin2* GCNs reduced costs by 72% from 20.80 to 5.73 USD and reduced the required time by 75% from 24 to 6 h.

The prevalence of CNVs of the *pfmdr1*, *pfplasmepsin2*, which are the molecular markers of mefloquine, piperaquine resistance respectively, and *pfgch1* linked to *pfdhfr/pfdhps* mutations which resulted in sulfadoxine-pyrimethamine resistance were assessed in Thai samples collected from year 2015–2019. The GCN results obtained with the ddPCR and qPCR assays were in 100% agreement. The proportion of isolates with amplified *pfmdr1* remains at low prevalence or zero in the two locations. This could be results of low drug pressure as mefloquine was discontinued as a national policy for treatment of uncomplicated falciparum malaria in Thailand since 2013. Since then piperaquine has been replaced [31]. The results of *pfplasmepsin2* gene amplification associated with piperaquine resistance were in agreement with previous publications [32, 33], which showed amplification of *pfplasmepsin2* in Northeast Thailand from 2011 to 2018. This suggested piperaquine resistance in *P. falciparum* is prevalent in Northeast Thailand.

Although sulfadoxine-pyrimethamine (SP) anti-malarial treatment was no longer used as a national policy for treatment of uncomplicated falciparum malaria in Thailand from since 1990, the results of the present study revealed substantial amplification of the *pfgch1* in Yala, near the Malaysian border. Persistence of high prevalence of antifolate resistance haplotypes in Thailand may be explained by several factors, such as continued drug pressure from non-malarial antifolate drugs such as trimethoprim and sulfamethoxazole [34], therefore, antifolate gene mutations/amplification might have been sustained because of continued presence of this antifolate drug pressure. The prevalence of the *pfmdr1*, *pfplasmepsin2* and *pfgch1* GCNs obtained from this study might be useful for surveillance of the efficacy of anti-malarial drugs.

## Conclusions

Uniplex, duplex and multiplex ddPCR assays for detection of the CNVs of the *P. falciparum mdr1*, *plasmepsin2* and *gch1* genes were developed and validated. The results confirmed the accuracy and precision of the proposed assays, which reduced the cost and turn-around time for surveillance of the efficacy of anti-malarial drugs. The assay is a valuable additional tool for genetic surveillance of anti-malarial drug resistance.

## Supplementary Information

**Additional file 1.** Primers and probes used for ddPCR and qPCR assays, and the optimal annealing temperature used.

**Additional file 2.** Two dimensional ddPCR amplitude plots of duplex ddPCR assays. The duplex ddPCR assay of *pfmdr1/pf-β-tubulin* plot (a.), duplex ddPCR assay of *pfplasmepsin2/pf-β-tubulin* plot (b.), and duplex ddPCR assay of *pfgch1/pf-β-tubulin* plot (c.) shows droplets with at least one copy of target genes (blue), reference gene (green), droplets contain both target and reference gene (orange), and empty droplets (grey).

**Additional file 3.** Genes copy number of *P. falciparum* reference strains estimated by ddPCR and qPCR assays.

**Additional file 4.** Genes copy number of *P. falciparum* isolated from Thailand.

## Data Availability

All data generated or analyzed during this study are included in this published article and its supplementary information files.

## Abbreviations

DNA: Deoxyribonucleic acid
ddPCR: Droplet digital PCR
GCN: Gene copy number
PCR: Polymerase chain reaction
18S rRNA: *18s ribosomal RNA* gene
*pfmdr1*: *P. falciparum multidrug resistance 1* gene
*pfgch1*: *P. falciparum GTP cyclohydrolase 1* gene
*pfplasmepsin2*: *P. falciparum plasmepsin2* gene
nM: Nanomolar
µL: Microliter
