# Supplementary material for: Measurement of gene amplifications related to drug resistance in Plasmodium falciparum using droplet digital PCR
Source: Malar J. 2021 Feb 28;20:120. doi: 10.1186/s12936-021-03659-5 (PMC7916280; doi:10.1186/s12936-021-03659-5)
Supplement: Supplementary file 1 — Additional file 1. Primers and probes used for ddPCR and qPCR assays, and the optimal annealing temperature used. [file 12936_2021_3659_MOESM1_ESM.pdf]

Table: Primers, probes, and optimal annealing temperature temperatures used for ddPCR assays [6, 7, 11].

No.	Methods	Genes	Primers and probe	DNA sequences	Optimum annealing temperature
1	Singleplex ddPCR of *pfmdr1*	*pfmdr1*	Forward	5'-TGCATCTATAAAACGATCAGACAAA-3'	56
			Reverse	5'-TCGTGTGTTCCATGTGACTGT-3'	
			Probe	FAM 5'-TTTAATAACCCTGATCGAAATGGAACCTTTG-3' TAMRA	
2	Singleplex ddPCR of *pfplasmepsin2*	*pfplasmepsin2*	Forward	5'-ATGGTGATGCAGAAGTTGGA-3'	56
			Reverse	5'-AACATCCTGCAGTTGTACATTTAAC-3'	
			Probe	FAM 5'-CAGGATCTGCTAATTTATGGGTCCCA-3'TAMRA	
3	Singleplex ddPCR of *pfgch1*	*pfgch1*	Forward	5'-CCTTTTGAAGGTACATGTGATATTGAGT-3'	56
			Reverse	5'-GCGTTACAAATATCGTTAGTTAAATCTTCT-3'	
			Probe	FAM 5'-CTTGAAAATTTAGATAACCCGA-3'TAMRA	
4	Singleplex ddPCR of *pf-β-tubulin*	*pf-β-tubulin*	Forward	5'-AAAAATATGATGTGCGCAAGTGA-3'	56
			Reverse	5'-AACTTCCTTTGTGGACATTCTTCCT-3'	
			Probe	VIC 5'-TAGCACATGCCGTTAAATATCTTCCATGTCT-3' TAMRA	
5	duplex ddPCR assay of *pfmdr1/pf-β-tubulin*	*pfmdr1*	Forward	5'-TGCATCTATAAAACGATCAGACAAA-3'	58
			Reverse	5'-TCGTGTGTTCCATGTGACTGT-3'	
			Probe	FAM 5'-TTTAATAACCCTGATCGAAATGGAACCTTTG-3' TAMRA	
		pf-β-tubulin	Forward	5'-AAAAATATGATGTGCGCAAGTGA-3'	
			Reverse	5'-AACTTCCTTTGTGGACATTCTTCCT-3'	
			Probe	VIC 5'-TAGCACATGCCGTTAAATATCTTCCATGTCT-3' TAMRA	
6	duplex ddPCR assay of *pfmdr1/pf-β-tubulin*	*pfplasmepsin2*	Forward	5'-ATGGTGATGCAGAAGTTGGA-3'	58
			Reverse	5'-AACATCCTGCAGTTGTACATTTAAC-3'	
			Probe	FAM 5'-CAGGATCTGCTAATTTATGGGTCCCA-3'TAMRA	
		pf-β-tubulin	Forward	5'-TGTGCGCAAGTGATCC-3'	
			Reverse	5'-TTTGTGGACATTCTTCCTC-3'	
			Probe	VIC 5'-CACATGCCGTTAAATATCTTCCATGTCT-3'TAMRA	
7	duplex ddPCR assay of *pfgch1/pf-β-tubulin*	*pfgch1*	Forward	5'-CCTTTTGAAGGTACATGTGATATTGAGT-3'	60
			Reverse	5'-GCGTTACAAATATCGTTAGTTAAATCTTCT-3'	
			Probe	FAM 5'-CTTGAAAATTTAGATAACCCGA-3'TAMRA	
		pf-β-tubulin	Forward	5'-AAAAATATGATGTGCGCAAGTGA-3'	
			Reverse	5'-AACTTCCTTTGTGGACATTCTTCCT-3'	
			Probe	VIC 5'-TAGCACATGCCGTTAAATATCTTCCATGTCT-3' TAMRA	

No.	Methods	Genes	Primers and probe	DNA sequences	Optimum annealing temperature
8	Multiplex ddPCR assay of *pfmdr1/pfplasmepsin2/pf-β-tubulin*	*pfmdr1*	Forward	5'-TGCATCTATAAAACGATCAGACAAA-3'	60
			Reverse	5'-TCGTGTGTTCCATGTGACTGT-3'	
			Probe	FAM 5'-TTTAATAACCCTGATCGAAATGGAACCTTTG-3' TAMRA	
		pfplasmepsin2	Forward	5'-ATGGTGATGCAGAAGTTGGA-3'	
			Reverse	5'-AACATCCTGCAGTTGTACATTTAAC-3'	
			Probe	FAM 5'-CAGGATCTGCTAATTTATGGGTCCCA-3'TAMRA	
		pf-β-tubulin	Forward	5'-AAAAATATGATGTGCGCAAGTGA-3'	
			Reverse	5'-AACTTCCTTTGTGGACATTCTTCCT-3'	
			Probe	VIC 5'-TAGCACATGCCGTTAAATATCTTCCATGTCT-3' TAMRA	
