# Supplementary figures and images for: Measurement of gene amplifications related to drug resistance in Plasmodium falciparum using droplet digital PCR
Source: Malar J. 2021 Feb 28;20:120. doi: 10.1186/s12936-021-03659-5 (PMC7916280; doi:10.1186/s12936-021-03659-5)

a.

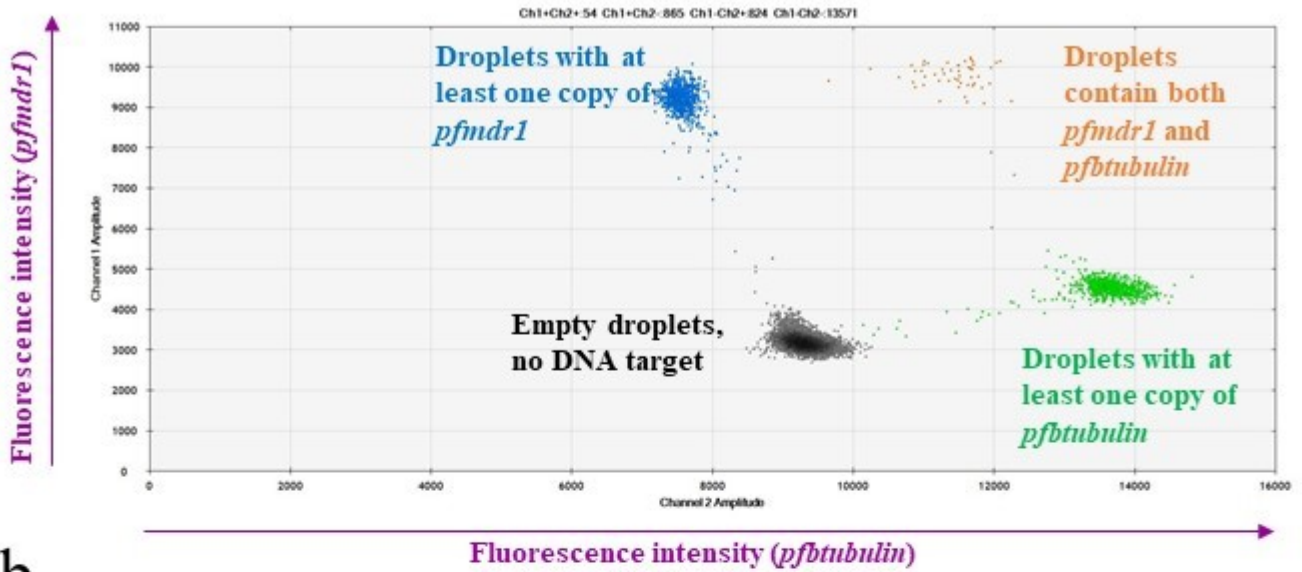

b.

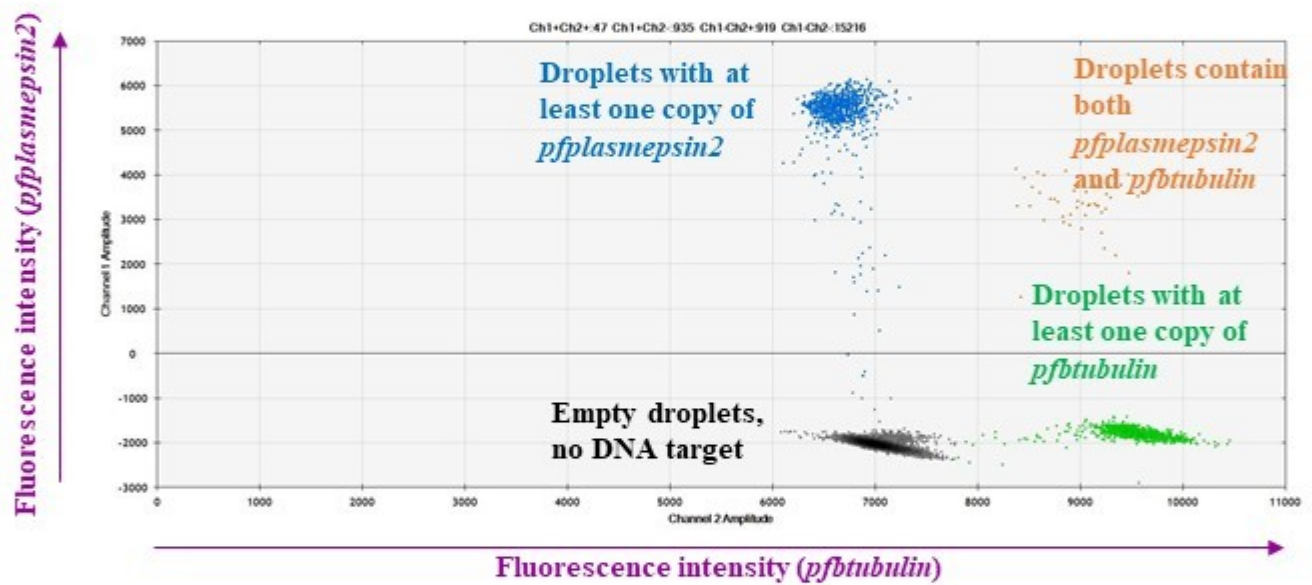

c.

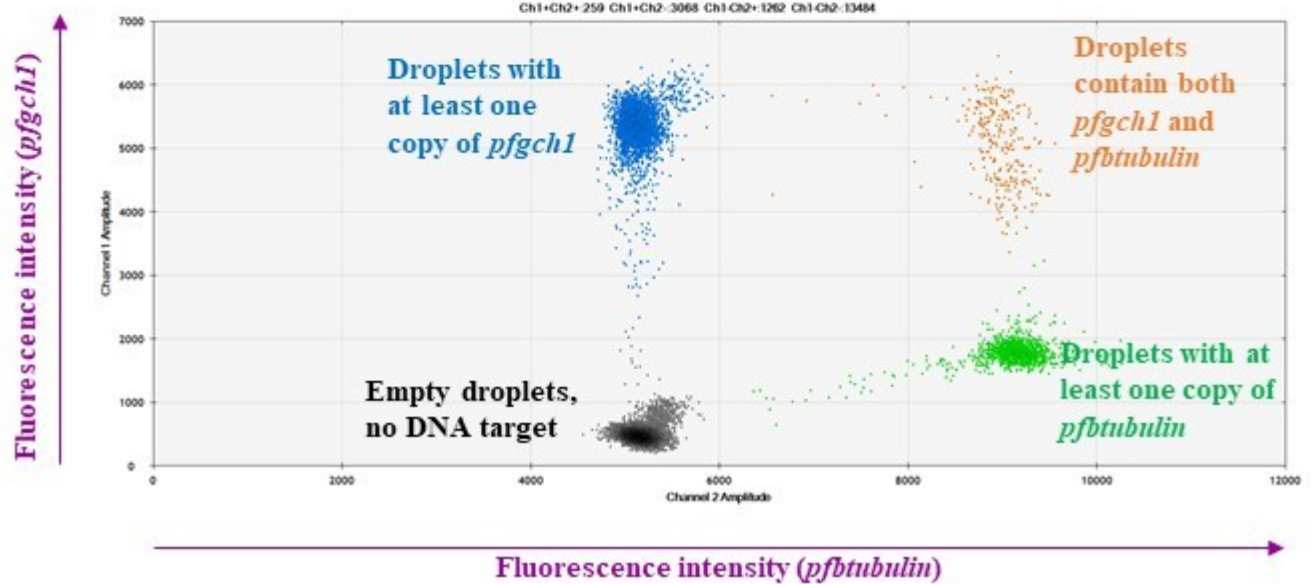

Supplement: Supplementary file 2 — Additional file 2. Two dimensional ddPCR amplitude plots of duplex ddPCR assays. The duplex ddPCR assay of pfmdr1/pf-β-tubulin plot (a.), duplex ddPCR assay of pfplasmepsin2/pf-β-tubulin plot (b.), and duplex ddPCR assay of pfgch1/pf-β-tubulin plot (c.) shows droplets with at least one copy of target genes (blue), reference gene (green), droplets contain both target and reference gene (orange), and empty droplets (grey). [file 12936_2021_3659_MOESM2_ESM.pdf]
